# Caseous Necrosis of Mitral Annulus

**DOI:** 10.1155/2015/561329

**Published:** 2015-08-18

**Authors:** Sinan Balci, Selcuk Akkaya, Selin Ardali, Tuncay Hazirolan

**Affiliations:** Department of Radiology, Hacettepe University Faculty of Medicine, Sıhhiye, 06100 Ankara, Turkey

## Abstract

Masses or mass-like lesions located in proximity to mitral valve encompass a wide range of differential diagnoses including neoplasias, abscesses, thrombi, and rarely caseous calcification of mitral annulus. Due to asymptomatic presentation, its diagnosis is usually incidental. Echocardiography is the first choice of imaging in evaluation. Cardiac computed tomography (CT) is helpful in establishing diagnosis by showing dense calcifications while cardiac magnetic resonance imaging (MRI) is used primarily as a problem solving tool. Imaging in evaluation of mitral annulus caseous calcification is essential in order to prevent unnecessary operations.

## 1. Introduction

Caseous calcification is a rare cause of mitral calcification, encountered more commonly in elder women. Since it is a benign process, differentiating it from malignant intracardiac masses precludes unnecessary surgical interventions. Various imaging modalities can be used for definitive diagnosis. Herein we present four cases of caseous calcification of mitral annulus (CCMA).

## 2. Case  1

**Figure 1:**
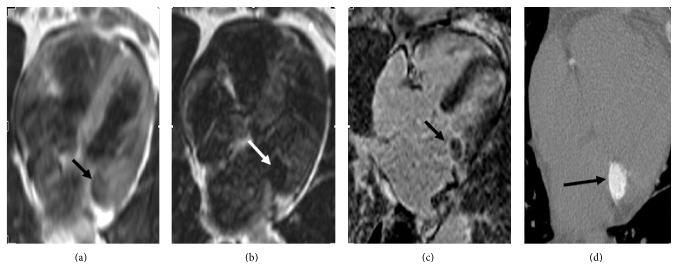
T1-weighted (a) and T2-weighted (b) turbo spin echo images show hypointense round mass-like lesion (black and white arrows). Delayed postcontrast cardiac MRI image (c) shows peripheral enhancement (black arrow). Multislice noncontrast computed tomography image (d) shows round calcification (black arrow) located in mitral annulus.

A 76-year-old male patient with symptoms of tachycardia, palpitation, sweating, and headache was admitted to our hospital. His history for coronary or cerebral atheroslerotic disease was not remarkable. Physical examination revealed diastolic murmur. The transesophageal echocardiography demonstrated hyperechoic mass in posterior leaflet of mitral valve and mild mitral regurgitation. There was not accompanying leaflet prolapse or mitral stenosis. Cardiac magnetic resonance imaging (MRI) was performed for further evaluation. It showed a hypointense mass-like lesion of 2.5 × 2 × 3 cm. Lesion was hypointense in both T1- and T2-weighted sequences and showed peripheral enhancement in postcontrast delayed series. The aforementioned MRI findings were suggestive of caseous necrosis (Figures 1(a)–1(c)). Multislice computed tomography (CT) showed a calcified mass, confirming presumptive diagnosis of caseous necrosis (Figure 1(d)).

## 3. Case  2

A 62-year-old female patient presented with chest pain and history of essential hypertension. Physical examination was unremarkable. Laboratory tests revealed no abnormality except minimal high level of LDL cholesterol. Multislice coronary artery CT angiography demonstrated caseous calcification of mitral annulus. CT examination also revealed atherosclerotic wall thickening and millimetric plaque formation in arcus aorta and descending aorta as well as approximately 50% stenosis in proximal left anterior descending artery.

## 4. Case  3

A 57-year-old female patient having a history of lupus nephritis was admitted to our hospital with complaints of shivering and fever. She has been undergoing dialysis for several years due to chronic renal failure. She did not have history of atherosclerotic vascular disease. On echocardiography, calcific mass-like lesion was noticed and, with respect to her clinical findings, infective endocarditis was considered as presumptive diagnosis and the patient was referred to radiology for further evaluation. The lesion's echocardiographic features were not suggestive of Libman-Sacks endocarditis. Thorax CT confirmed calcific nature of lesion and showed minute atherosclerotic plaques in coronary arteries and aorta. In order to document the exact nature of lesion located in mitral annulus, cardiac MRI was performed. MRI showed diffuse hypointensity in polypoid lesion in all sequences compatible with caseous calcification. Blood samples did not reveal any bacterial overgrowth suspicious for infective endocarditis.

## 5. Case  4

**Figure 2:**
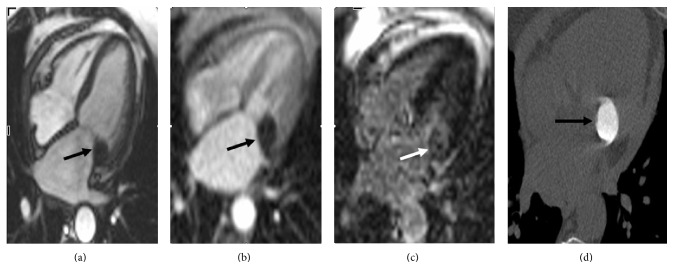
Balanced steady state free precession (SSFP) sequence image (a) shows hypointense lesion with well-defined borders (black arrow). First pass image (b) demonstrates no contrast enhancement. Delayed phase postcontrast phase sensitive inversion recovery (PSIR) image (c) shows peripheral enhancement. Multidetector cardiac CT image for calcium scoring (d) shows diffuse calcific lesion with well-defined borders located in mitral annulus (black arrow).

A 69-year-old female patient was referred to our institution with suspicion of cardiac mass in transesophageal echocardiography. Cardiac MRI performed in our hospital confirmed the mitral annulus location of mass and peripheral contrast enhancement in delayed postcontrast series (Figures 2(a)–2(c)). CT sections taken from only mitral annulus level showed dense calcification of lesion (Figure 2(d)).

## 6. Discussion

CCMA is rarely encountered in routine clinical practice, accounting for approximately 0.6% of all mitral calcifications. In different series, general prevalence is estimated to be 0.05% [1]. Patients with CCMA either are asymptomatic or present with irrelevant clinical symptoms. Spontaneous resolution of mitral annulus caseous calcification has been reported in the literature [2]. Exact underlying pathogenesis is not well understood; however elevated levels of blood calcium in patients undergoing dialysis have been proposed as an alternative mechanism [3]. Caseous calcification is microscopically composed of basophilic areas with fibrous tissue around it. Cartilage tissue may be rarely encountered; however bone formation is never seen [4].

Diagnosis is usually based on various imaging modalities. Echocardiography is the mainstay diagnostic technique. Typical echocardiographic findings include an echogenic, round, or occasionally semilunar mass with well-defined borders, located usually on the posterior mitral annulus [1]. A relatively hypoechoic central area is thought to represent liquefaction [5]. Cardiac CT and MRI also can be helpful in confirming or establishing diagnosis. On multislice CT, it is usually encountered as a mass with high density. As it is a diffuse calcified mass-like lesion, noncontrast CT or thorax CT imaging not dedicated to heart performed with a few slices encompassing mitral annulus region would be sufficient for definitive diagnosis. It does not enhance after IV contrast administration [6].

Cardiac MRI may be extremely helpful in equivocal cases as a problem solving tool. CCMA is seen as a hypointense lesion both in T1- and T2-weighted sequences [7]. In postcontrast studies, contrast enhancement on first pass sequences is not expected; however it may exhibit peripheral enhancement during delayed postcontrast sequences as seen in our second case.

Main differential diagnosis of CCMA includes cardiac neoplasms, endocarditis vegetations, abscesses, and thrombi located in left chambers. Cardiac heterogeneous mass lesions with accompanying local invasion and metastases favor the diagnosis of cardiac malignant neoplasm. In a septic patient or with a given clinical setting compatible with sepsis or widespread infection, presence of a mitral mobile mass should raise the suspicion of a vegetation or abscess [8]. Myxoma is mobile enhancing lesion and unlike caseous necrosis, it does not contain extensive calcification.

## 7. Conclusion

CCMA is a rarely encountered clinical entity; however it should be kept in mind in the differential diagnosis of intracardiac masses especially when located near mitral annulus. Although echocardiography is the mainstay imaging modality for diagnosis of CCMA, definitive diagnosis can be established via multidetector CT and cardiac MRI.
